# The effectiveness of disinfectant and steam exposure treatments to prevent the spread of the highly invasive killer shrimp, *Dikerogammarus villosus*

**DOI:** 10.1038/s41598-020-58058-8

**Published:** 2020-02-05

**Authors:** Stephanie J. Bradbeer, Neil E. Coughlan, Ross N. Cuthbert, Kate Crane, Jaimie T. A. Dick, Joe M. Caffrey, Frances E. Lucy, Trevor Renals, Eithne Davis, Daniel A. Warren, Benjamin Pile, Claire Quinn, Alison M. Dunn

**Affiliations:** 1 School of Biology, Faculty of Biological Sciences, University of Leeds, Leeds, LS2 9JT UK; 2 Institute for Global Food Security, School of Biological Sciences, Queen’s University Belfast, 19 Chlorine Gardens, Belfast, BT9 5DL Northern Ireland UK; 3 Queen’s Marine Laboratory, Queen’s University Belfast, 12-13 The Strand, Portaferry, BT22 1PF Northern Ireland UK; 4 Centre for Environmental Research, Innovation & Sustainability, Institute of Technology Sligo, Ash Lane, Co, Sligo, Ireland; 5 INVAS Biosecurity Ltd., 82 Lakelands Close, Stillorgan, Co., Dublin, Ireland; 6 Environment Agency, Sir John Moore House, Victoria Square, Bodmin, Cornwall, PL31 1EB UK; 7 School of Earth & Environment, University of Leeds, Leeds, LS2 9JT UK; 8 Water@leeds, School of Geography, University of Leeds, Leeds, LS2 9JT UK

**Keywords:** Freshwater ecology, Invasive species

## Abstract

Biosecurity protocols designed to prevent the spread of invasive alien species (IAS) are now an essential aspect of IAS management. However, the effectiveness of various biosecurity treatments requires further exploration. Killer shrimp, *Dikerogammarus villosus*, a notoriously high impact and ecosystem destabilising invader, has rapidly spread across Europe, and is of concern to invade Northern America. In this study, we examine the effectiveness of three commonly used, broad-spectrum disinfectants to cause mortality of *D. villosus*: Virasure Aquatic, Virkon Aquatic, and Virkon S. Immersion and spray treatments of 1%, 2% and 4% disinfectant solutions were examined for applications of up to 300 secs immersion and for up to ten consecutive sprays. Furthermore, we assessed the effectiveness of steam (≥100 °C) treatments for up to 120 secs. For all disinfectants, immersion in 1% solutions caused 100% mortality at ≥120 secs. At higher concentrations, shorter immersion times caused complete mortality: 60 and 15 secs for 2% and 4% solutions, respectively. Five sprays of 2% and 4% solutions resulted in 100% mortality, for all disinfectants. Direct steam exposure was highly effective, with complete *D. villosus* mortality occurring at ≥10 secs. Overall, brief exposure to broad-spectrum disinfectants and direct steam could be used to limit *D. villosus* spread.

## Introduction

Biological invasions of invasive alien species (IAS) are a major driver of biodiversity loss, and detrimentally affect the structuring, functioning, economic and social value of ecosystems worldwide^1,2^. In an age of rapid globalisation, the anthropogenic platforms that facilitate the accidental spread of IAS are well documented and the rate of biological invasions has increased in recent years^3^. Invasive alien species, synonymously termed invasive non-native species are species present outside of their native range with associated adverse impacts^4^. Given the multiple dispersal pathways and array of vectors^5–8^, freshwater ecosystems in particular are considered to be especially vulnerable to the introduction and further spread of IAS^2,9,10^. There is a wide range of users’ that access and work in the freshwater environment, all of which can present a risk of spreading IAS from one location to another. Management options for effective and efficient control and eradication of established IAS populations are often complex, expensive, resource-intensive, and can be damaging to non-target species^10,11^. Therefore, prevention of the introduction and secondary spread of IAS is the first line of defence. Biosecurity is the collectively term for actions taken to decontaminate equipment and thus aims to prevent the spread of IAS and has become a key aspect of IAS management strategies^12–14^. Accordingly, there is an urgent need to enhance biosecurity by identifying simple prevention protocols that minimise risk of spread yet remain user and environmentally friendly^13,15–18^.

Aquatic disinfectants such as Virasure Aquatic, Virkon S and Virkon Aquatic are used by recreational water users and responsible authorities, including government agencies, for the decontamination of equipment. These disinfectants are available in powder or tablet form and can be applied through spray applications or immersion of the equipment into disinfectant solutions. Although broad-spectrum aquatic disinfectants have been demonstrated to kill harmful pathogenic microbes and various invasive Mollusca species^19,20^, the effectiveness of these oxidising agents in killing free-living aquatic IAS requires further study. Recent studies have shown partial effectiveness of disinfectants in killing invasive aquatic plants and invertebrates^14,17,18^. However, identification of optimal disinfectant treatment to achieve complete mortality of a range of IAS, whilst minimising time and expense, including testing several types of disinfectants is still required. Steam exposure has also been proposed as a treatment to decontaminate equipment that may have been exposed to IAS. Short applications of steam have been effective in killing both invasive macrophytes^21^ and invertebrates^17,22,23^, however, further testing is still required to determine efficacy for species-specific treatments. Further, the identification of practical and efficacious biosecurity protocols is essential for the reliable uptake of biosecurity practices by environmental stakeholders and prevent behavioural barriers^15^.

The killer shrimp, *Dikerogammarus villosus* (Sowinsky 1894), is a highly invasive euryoecious amphipod crustacean native to the Ponto-Caspian region. It has spread and successfully colonised most of the major European inland waterways^1^ having been spread by many anthropogenic vectors^5,6^ and is of concern to invade Northern America. Capable of destabilising ecosystems, *D. villosus* is an especially damaging invader that causes profound declines of native macroinvertebrate populations^1,24^, and has been found to even prey upon fish eggs and larvae^25^. The propagule pressure associated with *D. villosus* is considered to be high, as one gravid female can to hold up to 190 eggs^26^, therefore introductions of even one organism may result in establishment, as seen in other amphipod species^27^. Accordingly, the need to prevent further potential spread, and therefore reduce propagule pressure of this prolific invader is clear.

In this study, we examined the efficacy of immersion and spraying of selected broad-spectrum disinfectants and direct steam exposure to cause mortality of *D. villosus*. To achieve this we examine the effectiveness of three commonly used disinfectants, including two previously untested disinfectants, Virasure Aquatic and Virkon Aquatic. In addition, we assess the effectiveness of direct steam spray for a range of application durations, including a very short and previously untested duration of 5 secs. Examined exposure times were designed to reflect realistic application times achievable by users of such biosecurity protocols. We hypothesise that greater concentrations and longer exposure times will cause substantial, if not complete mortality of *D. villosus* specimens, reducing potential propagule pressure. Equally, we expect that longer exposures of steam that induce thermal shock, rapidly killing *D. villosus*.

## Results

### Immersion in 1% disinfectant solutions

Immersion in 1% disinfectant caused significant mortality in *D. villosus* (χ^2^ = 432.32, df = 3, P < 0.001). Total mortality of *D. villosus* was evidenced following immersion in 1% of all three disinfectant solutions for ≥120 secs (Table 1). All control groups displayed 0% mortality. Furthermore, for 1% solutions, Virasure Aquatic caused significantly higher mortality than either Virkon Aquatic or Virkon S (both P < 0.05). For example, at 30 secs exposure, Virasure Aquatic resulted in >70% mortality compared to <50% mortality for both Virkon disinfectants. Exposure time also significantly affected mortality (χ^2^ = 107.71, df = 3, P < 0.001), wherein the percentage mortality following immersion of 30 secs was significantly lower than that of longer exposure times (all P < 0.001). There was no significant interaction term (χ^2^ = 9.16, df = 9, P > 0.05).

**Table 1:** Mean (±SE) raw percentage mortality of *Dikerogammarus villosus* at 24 hr following exposure to disinfectant and steam treatments. All treatments were replicated three times. Underlined and bold region delineates complete mortality.

Treatment	Concentration	Exposure Time (s)					
		5	15	30	60	120	300
*Immersion in 1% disinfectants*							
Control	0%	—	—	0	0	0	0
Virasure Aquatic	1%	—	—	83.3 ± 12	**100**	**100**	**100**
Virkon Aquatic	1%	—	—	46.6 ± 3.3	96.3 ± 3.3	**100**	**100**
Virkon S	1%	—	—	40 ± 5.7	86.6 ± 3.3	**100**	**100**
*Immersion in 2% and 4% disinfectants*							
Control	0%	0	0	3.3 ± 3.3	3.3 ± 3.3	—	0
Virasure Aquatic	2%	23.3 ± 8.8	**100**	**100**	**100**	—	**100**
	4%	46.6 ± 8.8	**100**	**100**	**100**	—	**100**
Virkon Aquatic	2%	3.3 ± 3.3	56.7 ± 12	**100**	**100**	—	**100**
	4%	23.3 ± 8.8	**100**	**100**	**100**	—	**100**
Virkon S	2%	3.3 ± 3.3	56.7 ± 26	86.6 ± 3.3	**100**	—	**100**
	4%	80 ± 15.3	**100**	**100**	**100**	—	**100**
		**No. of sprays**					
		**2**		**5**		**10**	
*Disinfectant spray*							
Control	0%	0		0		6.6 ± 6.6	
Virasure Aquatic	1%	66.6 ± 24		80 ± 20		**100**	
	2%	20 ± 11.5		**100**		**100**	
	4%	**100**		**100**		**100**	
Virkon Aquatic	1%	20 ± 11.5		66.6 ± 13.3		86.6 ± 13.3	
	2%	13.3 ± 13.3		**100**		**100**	
	4%	80 ± 20		**100**		**100**	
Virkon S	1%	6.6 ± 6.6		73.3 ± 13.3		86.6 ± 6.6	
	2%	33.3 ± 13.3		**100**		**100**	
	4%	**100**		**100**		**100**	
		**Exposure Time (s)**					
		**Control**	**5**	**10**	**30**	**60**	**120**
*Steam spray*							
Steam ≥ 100 °C	—	0	70 ± 25.2	**100**	**100**	**100**	**100**

### Immersion in 2% or 4% disinfectant solutions

Following immersion treatments in 2% and 4% disinfectant solutions, total *D. villosus* mortality was observed for all disinfectant treatments at exposure durations of ≥60 secs (Table 1). A low mean mortality rate of 3.3% was detected for the control groups. Overall, treatment had a significant effect on *D. villosus* mortality (χ^2^ = 712.59, df = 6, P < 0.001). Treatment with 2% Virasure Aquatic was significantly more effective than either 2% Virkon Aquatic or Virkon S (both P < 0.001). Furthermore, immersions in all 4% disinfectant solutions were significantly more efficacious than 2% disinfection in Virkon Aquatic or Virkon S (all P < 0.001). However, submersion in 2% Virasure Aquatic did not differ significantly from 4% Virasure Aquatic or 4% Virkon Aquatic (both P > 0.05), whilst 4% Virkon S was more effective than 2% Virasure Aquatic (P < 0.001). Immersion in 4% Virkon S was significantly more effective in inducing *D. villosus* mortality than 4% Virkon Aquatic (P < 0.001), yet was more similar to 4% Virasure Aquatic (P > 0.05). Exposure time significantly affected mortality (χ^2^ = 499.42, df = 4, P < 0.001), with 5 secs and 15 secs exposures significantly less efficacious than 30 secs, 60 secs or 300 secs exposures overall (all P < 0.01). There was no significant interaction term (χ^2^ = 16.52, df = 24, P > 0.05).

### Disinfectant spray

Disinfectant spray treatments caused significant mortality of *D. villosus* (χ^2^ = 247.43, df = 9, P < 0.001). A low mean mortality rate of up to 6.6% was recorded within control groups (Table 1). Total *D. villosus* mortality was observed following treatments of 2% and 4% solutions of all three disinfectants after 5 sprays. Five spray treatments of 1% solutions resulted in high but not complete mortality. The maximum number of sprays tested here, 10 sprays, resulted in a mean mortality of 86.6% for 1% Virkon Aquatic and Virkon S and 100% mortality for 1% Virasure Aquatic. Treatment with 1% Virasure Aquatic caused greater mortality rates than either 1% Virkon Aquatic or 1% Virkon S solutions (both P < 0.05), whilst the two Virkon products were more similar (P > 0.05). All 4% disinfectant treatments caused significantly greater mortality than 1% Virkon Aquatic or 1% Virkon S solutions (all P < 0.01), but were more similar to 1% Virasure Aquatic (all P > 0.05). Treatments with 2% disinfectants were similar among products (all P > 0.05). Increased quantity of sprays also significantly influenced mortality (χ^2^ = 140.99, df = 2, P < 0.001), with mortality following 2 sprays being significantly lower than treatment with 5 or 10 sprays, at all concentrations (all P < 0.001). There was no significant interaction term between spray treatment and exposure (χ^2^ = 5.83, df = 18, P > 0.05).

### Steam spray

Total *D. villosus* mortality was caused by direct steam exposures of ≥10 secs, whilst exposure for 5 secs resulted in mean 70% mortality (Table 1). All control groups displayed 0% mortality. Steam treatments had a significant effect on mortality of *D. villosus* (χ^2^ = 148.13, df = 5, P < 0.001). There were no significant differences in mortality among steam application durations (all P > 0.05).

## Discussion

Immersion in disinfectant solutions was shown to be a suitable potential biosecurity treatment leading to complete *D. villosus* mortality. Mortality was greater at higher concentrations of disinfectant and for longer immersion durations. For all three disinfectants tested, total mortality of *D. villosus* was achieved following immersion times of ≥120 secs, 60 secs and 15 secs for 1%, 2% and 4% solutions, respectively. Disinfectant spray treatments were also effective. Total *D. villosus* mortality was observed for all three disinfectants at 2% and 4% solutions following 5 spray treatments. High mortality (>85%) was recorded following 10 spray treatments of 1% solutions. Overall, for shorter immersion times and reduced spray exposure, Virasure Aquatic solutions appeared to be marginally more effective than Virkon S and Virkon Aquatic. Steam exposure was highly efficacious, with complete mortality occurring at exposure durations of ≥10 secs and high mortality (70%) at 5 secs exposure.

*Dikerogammarus villosus* can adhere and remain attached to water users’ equipment^28^, upon which they are capable of surviving for up to 16 days in damp conditions^12^. To inhibit the further overland spread of this highly invasive amphipod, biosecurity practices utilising disinfectant treatments would be especially beneficial for decontamination of small items of PPE and equipment. For instance, wetsuits, waders and nets could be completely immersed in disinfection baths, while spray applications may be more suitable for the decontamination of larger equipment, e.g. boats, outboard motors, and vehicles^21,29^. Furthermore, water intake-systems, designed to aid cooling of outboard motors, or large pipes such as those used in flood management and raw water movement could also be flushed with disinfectant solutions.

Whilst disinfectants have been shown to be effective against microbes and certain IAS^18–20^, evidence presented in the literature does indicate limited effectiveness against other IAS (e.g. macrophytes^16,17^). Our findings are in accordance with Sebire *et al*. (2018) who found 100% mortality of *D. villosus* after immersion in 1% Virkon S for 15 mins^18^. Previous research has identified treatment time as a barrier to good biosecurity practice^15^. We demonstrate that complete mortality can be achieved with shorter treatment durations (≥120 secs) using 1% disinfectant solutions, making this treatment potentially a useful addition to field biosecurity measures. Furthermore, we demonstrate that other disinfectants are also equally as effective as Virkon S, with equal application times of 1% solutions needed to achieve complete mortality of *D. villosus*.

We also found that short duration exposure to steam was effective against *D. villosus*, identifying 10 second exposure of steam resulting in complete mortality, in line with findings by Stebbing and Rimmer (2013)^22^. Direct application of pressurised jets of steam may prove to be highly beneficial, particularly when combined with additional cleaning methods such as hand removal, brushing or scraping^21,30^. Equally, steam treatments may aid decontamination of equipment items that are problematic to manually clean, such as niche areas or large complex structures like chain lockers, intake grates, pipework, trailers and vehicles. Whilst the effectiveness of steam to kill a number of IAS has been tested^21,23,31^, the application of steam needs to be tested against a wider range of IAS. Furthermore, considering the apparent effectiveness of thermal shock, pressurised hot water sprays should also be assessed in future studies against a range of IAS, including previously untested macrophytes^32,33^.

The provision of in-field biosecurity stations for a range of stakeholders could act as a suitable mechanism to limit IAS spread^15,21,31,34^. Installation of decontamination apparatus and facilities, with clear guidance, may facilitate utilisation of these simple but highly efficacious biosecurity techniques^13,34^. The provisioning of these biosecurity stations will also create a platform for raising awareness of IAS and biosecurity with all users. These biosecurity facilities could be placed at points of exit and entry (e.g. angling stations and boat ramps) to ensure ease of access to the steam cleaners or large soaking stations containing disinfectants^21,23,31^. Maintenance and responsibility of such stations would be essential, especially in the case of disinfectants, as the disinfectant solutions decay over time and become less effective. Furthermore, suitable disposal methods would need to be in place, such as interceptors for treatment water/disinfectant run-off, especially when considering equipment being cleaned prior to entering a site. Although the risk of toxicity to non-target aquatic organisms through disinfectant residues and spills is considered to be low^19^, further examination of low concentration lethality on non-target species is needed. This is of particular importance at biosecurity stations locations where there is a greater opportunity for repeated spills compared to a single-visited area. Biosecurity guidance must highlight the correct disposal of used disinfectant water. Furthermore, the legal issues concerning the use of broad-spectrum disinfectants as biosecurity agents for invasive macroscopic organisms will need to be addressed (e.g. herbicide or insecticide^16,18^).

The results presented here demonstrate that broad-spectrum disinfectants and direct steam applications could be used as part of effective and efficient biosecurity protocols to prevent the further anthropogenic-mediated spread of *D. villosus*. Reducing the propagule pressure of this prolific invader is essential. Accordingly, promotion and adoption of these techniques by biosecurity campaigns, stakeholder groups, and practitioners should be encouraged. Furthermore, the requirement to perform and adhere to a biosecurity standard should be incorporated into relevant Codes of Practice, with subsequent enforcement in relation to all water users.

## Methods

### Specimen collection and maintenance

*Dikerogammarus villosus* specimens were collected from Grafham Water, Cambridgeshire, UK (52°17′31.2″N, 0°19′23.9″W) and Cardiff Bay (51°27′14.7″N, 3°09′50.4″W). Specimens were transported in source water to the University of Leeds, UK and housed in aerated aquaria filled with dechlorinated tap-water, at a constant temperature of 14 °C under a 12:12 hr light-dark regime. Organisms were acclimated for over one week prior to experimental use. Adult specimens were used as they have been shown to be less susceptible to treatments than juveniles^18^. Specimens from Cardiff Bay were only used for the assessment of Virkon S disinfectant spray treatments, due to a shortage of Grafham Water specimens.

### Immersion in disinfectant solutions

The efficacy of three commercially available disinfectant products, Virasure Aquatic (Fish Vet Group), Virkon Aquatic and Virkon S (Antec Int. DuPont), was examined using 1% (10 g L^−1^), 2% (20 g L^−1^), or 4% (40 g L^−1^) disinfectant solutions, and a 0% (0 g L^−1^) control. For all disinfectants, the concentration recommended for general use against microbes is 1%, and we focused on this in addition to higher concentrations of 2 and 4%. All solutions were made using aerated dechlorinated tap water. Initially, immersion in 1% disinfectant solutions was assessed for four exposure times: 30 secs; 60 secs; 120 secs; 300 secs (*n* = 3 per experimental group). Following this, immersion in 2% and 4% solutions were separately assessed for five exposure times: 5 secs; 15 secs; 30 secs; 60 secs; 300 secs (*n* = 3 per experimental group).

In all cases, groups of ten *D. villosus* were weighed (mean ± SE individual specimen weight: 115.0 ± 0.9 mg) and briefly maintained (<30 mins) in aerated dechlorinated tap water in circular containers (SA, 548 mm^2^; volume, 1917 mm^3^) prior to experimentation. Only active individuals that responded to a stimuli were selected; specimens that displayed visible parasitism or had recently moulted were not used. Using fine-meshed flat-bottomed sieves (SA, 528 mm^2^; volume, 1848 mm^3^), treatment groups were immersed in disinfectant solutions for the allotted exposure time. Control groups were likewise immersed in dechlorinated tap water (i.e. 0% solution) for the same exposure times. Following experimental exposure, the fine-mesh sieves containing the ten *D. villosus* were removed from the experimental solution and re-immersed in dechlorinated tap water for a two-minute period to remove excess disinfectant; this was repeated twice (see Cuthbert *et al*.^17^). Following this washing process, specimen groups were returned to 200 ml of aerated dechlorinated tap water in their original containers for a 24 hr recovery period (14 °C; 12:12 hr light-dark), after which mortality was assessed. Specimens were considered dead if they did not respond to stimuli and did not hold their pereopoda under their body^13^.

### Disinfectant spray

Mist-spray applications for all three disinfectants were examined using 1%, 2% or 4% solutions, and a 0% control. Groups of five *D. villosus* were weighed (107.4 ± 1.1 mg) and briefly maintained in fine-meshed flat-bottomed sieves (SA, 528 mm^2^; volume, 1848 mm^3^) within a circular container (SA, 548 mm^2^; volume, 1917 mm^3^) in dechlorinated tap water (<30 mins). The sieve was removed from the water and, using a hand-held spray bottle, 2, 5 or 10 spray applications of a disinfectant solution were delivered (*n* = 3 per experimental group). This were directly applied to treatment groups held within sieves, at a distance of 6–8 cm from the exit-point of the spray bottle. Application of one spray equated to 0.75 ml of solution per 528 mm^2^. The sieves containing the experimental specimens were then left air-exposed for a five minutes period (~20 °C), before being re-immersed in dechlorinated tap water for a period of two minute to removed excess disinfectant. This washing process was repeated twice. Following this, specimen groups were returned to 200 ml of aerated dechlorinated tap water and mortality was assessed following a 24 hr recovery period (as above).

### Steam spray

Specimens were directly exposed to a continuous jet of steam for 5 secs, 10 secs, 30 secs, 60 secs, and 120 secs (≥100 °C; Karcher SC3) (*n* = 3 per experimental group). Groups of ten *D. villosus* were weighed (111.5 ± 2.4 mg) and briefly maintained in fine-meshed flat-bottomed sieves (SA, 528 mm^2^; volume, 1848 mm^3^) in aerated dechlorinated tap water within a larger container (SA, 548 mm^2^; volume, 1917 mm^3^) prior to experimentation. Steam was directly applied to groups held within sieves at a distance of 6–8 cm from the exit-point of the lance, ensuring equal application over the surface area of the sieve. Following exposure, groups were air-exposed for a 10 minute period (~20 °C) to allow gradual cooling before being re-immersed in dechlorinated tap water. This was to avoid a second thermal shock occurring if specimens were immediately returned to water after exposure to high temperatures. Control groups were air-exposed for twelve minutes, i.e. the duration of the longest steam exposure and cooling period combined. Mortality was assessed following a 24 hr recovery period (as above).

### Statistical analysis

Statistical analyses were performed using R v3.5.1^35^. Mortality of *D. villosus* was analysed using generalised linear models (GLMs) assuming a binomial error distribution and logit link. Reduced-bias estimation and inference was used to account for complete separation^36^. Likelihood ratio tests via analysis of deviance were used to obtain effect sizes^37^ and post hoc tests were performed using least-square means with Tukey adjustments to account for multiplicity^38^, with α ≤ 0.05. First, the effects of immersion in 1% disinfectant solutions (4 levels: control; 1% Virasure Aquatic; 1% Virkon Aquatic; 1% Virkon S) and exposure time (4 levels: 30 secs; 60 secs; 120 secs; 300 secs) were collectively analysed. Second, the effects of immersion in both 2% and 4% disinfectant treatments (7 levels: control; 2%, 4% Virasure Aquatic; 2%, 4% Virkon Aquatic; 2%, 4% Virkon S) and exposure time (5 levels: 5 secs; 15 secs; 30 secs; 60 secs; 300 secs) on mortality rates were likewise assessed. In all cases, non-significant terms and interactions were removed stepwise to obtain the minimum adequate model. Similarly, mortality rates following disinfectant spray treatments were examined using GLMs with respect to spray treatment (10 levels: control, 1%, 2%, 4% Virasure Aquatic; 1%, 2%, 4% Virkon Aquatic; 1%, 2%, 4% Virkon S) and exposure time (3 levels: 2 sprays; 5 sprays; 10 sprays). Finally, the mortality rates of *D. villosus* following steam treatments were analysed using a GLM (6 levels: control; 5 secs; 10 secs; 30 secs; 60 secs; 120 secs).

## Data Availability

The datasets generated during and analysed during this study are available from the corresponding author on reasonable request.
